# Dual-IR late gadolinium enhancement achieves better blood suppression than traditional IR in a swine model of atrial radiofrequency ablation scar

**DOI:** 10.1186/1532-429X-15-S1-O89

**Published:** 2013-01-30

**Authors:** Sarah A Peel, James Harrison, Anne Yoon Krogh Grøndal, Lars Bloch, Esben S Hansen, Won Yong Kim, Steen Fjord, Rene M Botnar, Henrik Jensen, Reza Razavi, Mark O'Neill, Tobias Schaeffter

**Affiliations:** 1 Imaging Sciences and Bioengineering, King's College London, London, UK; 2 Department of Cardiology, Guy's and St. Thomas' NHS Foundation Trust, London, UK; 3 Department of Clinical Medicine, Aarhus University, Aarhus, Denmark; 4 Department of Cardiology, Aarhus University Hospital Skejby, Aarhus, Denmark; 5 MR-center, Aarhus University Hospital Skejby, Aarhus, Denmark

## Background

A good correlation has been found between the extent of late gadolinium enhancement (LGE) and clinical outcome in patients who have undergone radiofrequency (RF) ablation for atrial fibrillation (AF). Residual blood signal with the traditional inversion-recovery (IR) sequence often hampers scar visualization in these patients and causes poor repeatability of scar size measurements. The dual-IR sequence has previously been shown to improve blood suppression in LGE images of myocardial infarction. We sought to assess whether the superior blood suppression with the dual-IR sequence would improve atrial scar definition in a swine model of chronic atrial radiofrequency ablation scar.

## Methods

A 30 kg female Göttingen minipig pig underwent linear RF ablation from the SVC to the IVC under general anesthesia. MR imaging was performed two months later using a 1.5T MR scanner (Philips Healthcare, the Netherlands). After administering 0.2 ml/kg of Gadovist (Bayer Schering, Berlin), dual-IR imaging (imaging every heartbeat) was performed at 10 and 15 minutes and compared with standard IR imaging (imaging every 2 heartbeats) at 35 minutes.

The dual-IR pre-pulse consists of two non-selective inversion pre-pulses separated by two time delays (TI1 and TI2). The TI1 and TI2 delays were optimized to achieve signal suppression in the T1-range of 250-1400 ms. Whereas the IR sequence can only null one T1 species (e.g. normal myocardium), the dual-IR pre-pulse simultaneously suppresses both the blood and normal myocardium whilst maintaining high signal in the scar.

## Results

**Figure 1:**
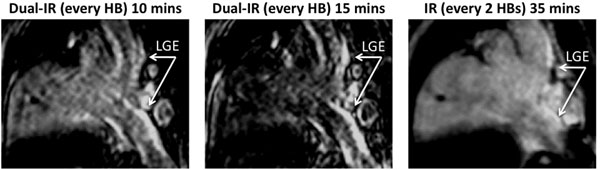
LGE images using the dual-IR sequence (10 and 15 minutes) and the IR sequence (35 minutes). Heart-rate was 70bpm. All images have identical windowing.

**Figure 2:**
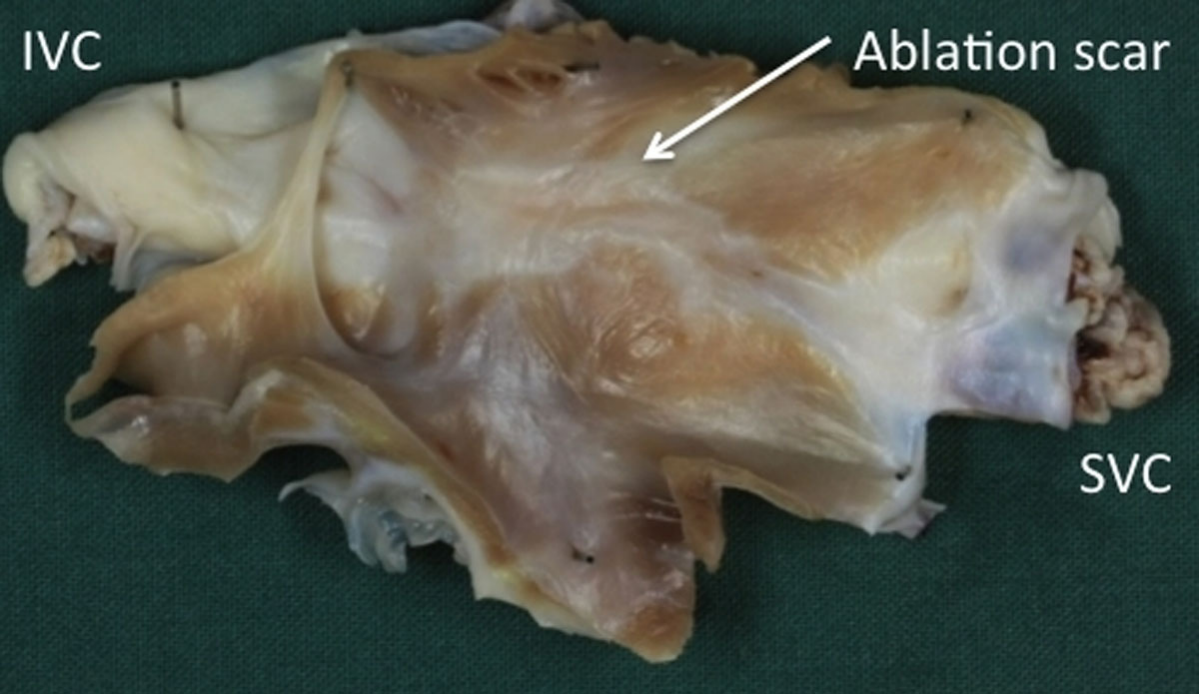
Image of chronic ablation scar on excised atrial tissue

Dual-IR images achieved superior blood suppression at an earlier time point compared with IR images (Figure 1). The enhancement corresponds well to the areas of scar shown on excised tissue (Figure 2).

## Conclusions

The dual-IR technique improves blood signal suppression and definition of the edges and boundaries of LGE areas. As dual-IR images were performed every heartbeat and taken earlier after contrast administration, it also has the potential to reduce the overall scan time.

## Funding

This work was funded by the British Heart Foundation award RE/08/003 and EU COST Action: TD1007. The authors also acknowledge financial support from the Department of Health via the National Institute for Health Research (NIHR) comprehensive Biomedical Research Centre award to Guy's & St Thomas' NHS Foundation Trust in partnership with King's College London and King's College Hospital NHS Foundation Trust and the Centre of Excellence in Medical Engineering funded by the Wellcome Trust and EPSRC under grant number WT 088641/Z/09/Z.

